# Sex differences in brain cell-type specific chromatin accessibility in schizophrenia

**DOI:** 10.21203/rs.3.rs-4158509/v1

**Published:** 2024-04-04

**Authors:** Panos Roussos, Yixuan Ma, Kiran Girdhar, Gabriel Hoffman, John Fullard, Jaroslav Bendl

**Affiliations:** Icahn School of Medicine at Mount Sinai; Icahn School of Medicine at Mount Sinai; Icahn School of Medicine at Mount Sinai; Icahn School of Medicine at Mount Sinai; Icahn School of Medicine at Mount Sinai; Icahn School of Medicine at Mount Sinai

## Abstract

Our understanding of the sex-specific role of the non-coding genome in serious mental illness remains largely incomplete. To address this gap, we explored sex differences in 1,393 chromatin accessibility profiles, derived from neuronal and non-neuronal nuclei of two distinct cortical regions from 234 cases with serious mental illness and 235 controls. We identified sex-specific enhancer-promoter interactions and showed that they regulate genes involved in X-chromosome inactivation (XCI). Examining chromosomal conformation allowed us to identify sex-specific *cis-* and *tra*ns-regulatory domains (CRDs and TRDs). Co-localization of sex-specific TRDs with schizophrenia common risk variants pinpointed male-specific regulatory regions controlling a number of metabolic pathways. Additionally, enhancers from female-specific TRDs were found to regulate two genes known to escape XCI, *(XIST* and JPX), underlying the importance of TRDs in deciphering sex differences in schizophrenia. Overall, these findings provide extensive characterization of sex differences in the brain epigenome and disease-associated regulomes.

## Introduction

In schizophrenia (SCZ), multiple clinical features, including age of onset, symptomatology, prevalence, the course of illness, and response to treatment often differ by sex^1–3^. Men are slightly more likely to develop SCZ than women and women tend to be older when they first experience symptoms. These differences have been ascribed to different gene contexts within the sex chromosomes, roles of hormones, cultural and environmental differences, and differences in behavior^4^. However, the molecular mechanisms and underlying biology remain largely unclear. A previous large-scale transcriptome study by the Genotype-Tissue Expression (GTEx) project^5^ characterized widespread differences in gene expression levels and identified 13,294 sex-differentially expressed genes across 44 human tissues^6^. Studies focusing specifically on the brain have identified differences in brain region-specificity^7^, developmental stages^8^, as well as the interaction between sex and complex traits^9,10^. In the context of disease, investigations into sex-specific effects on SCZ have primarily been limited to studies of the transcriptome^9,11,12^. Despite these valuable resources, the representation of sex-specific regulatory regions, particularly enhancers and promoters, is lacking and, as such, the interaction effects of sex and disease are under-characterized. While evidence from studies of both humans^13^ and mice^14^ indicate that sex-biased gene expression may arise from differences in chromatin accessibility, neither of these studies examined the brain.

In this study, we focused on sex- and disease-specific alterations in chromatin accessibility and interrogated the higher-order chromatin structure spanned by *cis-* and *trans-regulatory* domains (CRDs and TRDs). We generated and analyzed ATAC-seq data from 469 individuals (172 females, 297 males), in two broad cell types (neurons and non-neurons). We used this dataset to quantify and characterize sex differences in chromatin accessibility (sex-specific chromatin accessibility) and to identify sex-specific open chromatin regions (OCRs) associated with the regulation of genes involved in X-chromosome inactivation (XCI). Integrating these functional epigenomic data with common genetic risk variation allowed us to identify genetic effects on the epigenome that vary between sexes and colocalize with complex trait associations. Finally, by integrating disease-specific chromatin accessibility, we identified SCZ-associated OCRs within sex-specific TRDs, particularly those on the X chromosome involved in regulating XCI in the human brain. By combining our findings with functional gene sets and transcription factor binding annotation, we characterized both cell-type-specific and universal drivers and mechanisms of sex differences in the brain epigenome.

## Results

### Sex effects on chromatin accessibility

**Figure 1:**
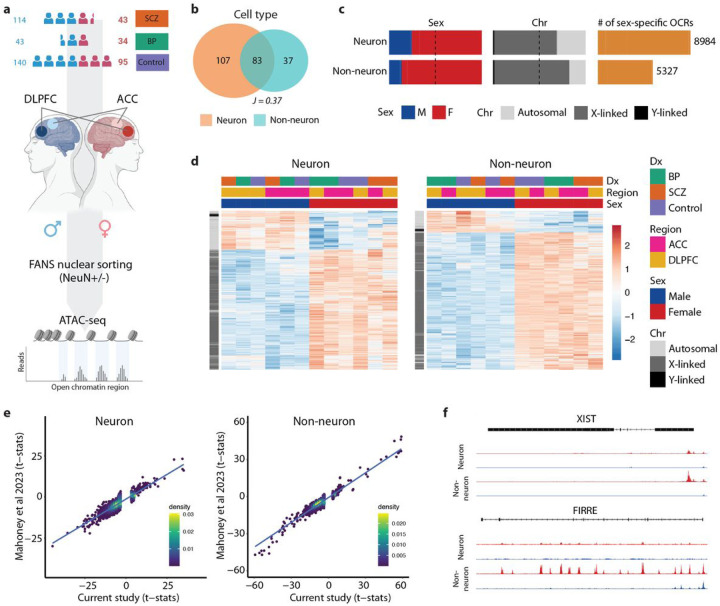
Sex-specific chromatin accessibility analysis in the human brain. **a)** Schematic illustration of the study design. ATAC-seq was performed on neuronal (NeuN+) and non-neuronal (NeuN−) nuclei isolated from two brain regions (DLPFC and ACC). **b)** Venn plot by cell type depicting the overlap in megabases of OCRs. *J* indicates the Jaccard index between the respective sets of OCRs. **c)** Bar plots depicting proportions of sex-specific OCRs stratified by sex (left panel), by chromosome (middle panel), and the total number of sex-specific OCRs (right panel) in neuronal and non-neuronal cells, respectively. M:Male, F:Female. **d)** Heatmaps depicting sex-specific neuronal (left panel) and non-neuronal (right panel) OCRs. The color-coded annotation layers above the heatmaps indicate groups of samples stratified by diagnosis (Dx), brain region (Region), and sex (Sex). The annotation layer to the left of the heatmaps shows sex-specific OCRs clustered by chromosome (Chr). The color intensity indicates t-statistics of differential chromatin accessibility, with red indicating upregulation and blue indicating downregulation. **e)** Scatter plots showing the correlation of t-statistics for male-vs-female comparison between the current study and the study by Mahoney et al.^20^ for neurons (left panel) and non-neurons (right panel), respectively. Each point represents an OCR, with the position along the x-axis indicating the t-statistic from the current study and the y-axis representing the t-statistic from Mahoney et al. **f)** Examples of sex-specific changes in chromatin accessibility of XCI genes.

We previously studied cell-type specific chromatin structure and genetic regulation of the brain regulome as part of the CommonMind Consortium (CMC)^15,16^. Here, we present an extensive characterization of sex differences in the human brain epigenome from 469 individuals (172 females, 297 males), consisting of 157 SCZ cases, 77 bipolar disorder (BD) cases, and 235 unaffected controls from the CMC dataset (Fig. 1a and Table S1). We performed the assay for transposase-accessible chromatin followed by sequencing (ATAC-seq) to quantify and characterize sex differences in chromatin accessibility in neuronal (NeuN+) and non-neuronal (NeuN-) nuclei isolated by fluorescence-activated nuclear sorting (FANS) from two brain regions, i.e. the anterior cingulate cortex (ACC) and dorsolateral prefrontal cortex (DLPFC). After a comprehensive quality control analysis of ATAC-seq libraries (Methods), the final dataset consisted of a total of 1,393 samples comprising over 54.8 billion unique reads (39.4 million reads per library) (Table S1). Due to the large chromatin accessibility differences between the two cell types (Fig. 1b), we separated neuronal and non-neuronal samples for downstream analysis. A total of 391,420 and 260,431 neuronal and non-neuronal open chromatin regions (OCRs) were then identified, respectively.

We next quantified sex-specific chromatin accessibility in each cell type. For each cell type, we first fit a linear mixed model using *dream*^17,18^ to account for covariates, including diagnosis, cell type, brain region, brain bank, as well as other technical and biological factors (Methods). Consequently, we discovered a total of 8,984 and 5,327 differentially accessible OCRs between males and females (sex-specific OCRs) in neuronal and non-neuronal cells, respectively (FDR < 0.05) (Fig. 1c and Table S2). We identified more female-specific OCRs than male-specific OCRs in both neuronal and non-neuronal cells, the majority of which mapped to the sex chromosomes (Fig. 1c, d). In neurons, 6,072 differentially accessible OCRs (68%) were X-linked, 132 (1%) were Y-linked, and 2,780 (31%) were autosomal; while in non-neurons, 4,305 OCRs (81%) were X-linked, 84 (1%) were Y-linked, and 938 (18%) were autosomal. As expected, the analysis of sex differences also revealed that all Y-linked regulatory elements with increased chromatin accessibility were specific to males, while nearly all X-linked regulatory elements with increased chromatin accessibility were specific to females, potentially due to escape from X-chromosome inactivation (XCI)^19^ (Table S4). By validating our differential chromatin accessibility analysis against another epigenome study of postmortem brain samples from DLPFC region^20^, we achieved a high concordance (Spearman correlation coefficients (ρ) of 0.877 and 0.869, p-value < 2.2e-16 for both) in neuronal and non-neuronal samples (Fig. 1e).

Following the observed enrichment of female-specific OCRs on the X chromosome, we next investigated sex-specific changes in chromatin accessibility patterns associated with XCI genes. Out of 277 genes known to escape XCI^6,19,21–25^, a substantial number – 205 genes in neurons and 206 genes in non-neurons (181 genes shared between the two cell types) – appear to be regulated by sex-specific OCRs (Table S3). Notably, *XIST*, a non-coding RNA essential for the initiation of XCI^26^, was found to be up-regulated by female-specific OCRs in neurons and non-neurons (Fig. 1f). *FIRRE*, an X-linked lncRNA that escapes XCI^27^, also demonstrated up-regulation in both cell types (Fig. 1f). This complex regulation of XCI highlights the sex-specific interplay between chromatin accessibility and gene expression.

### Functions of sex-specific chromatin accessibility

**Figure 2:**
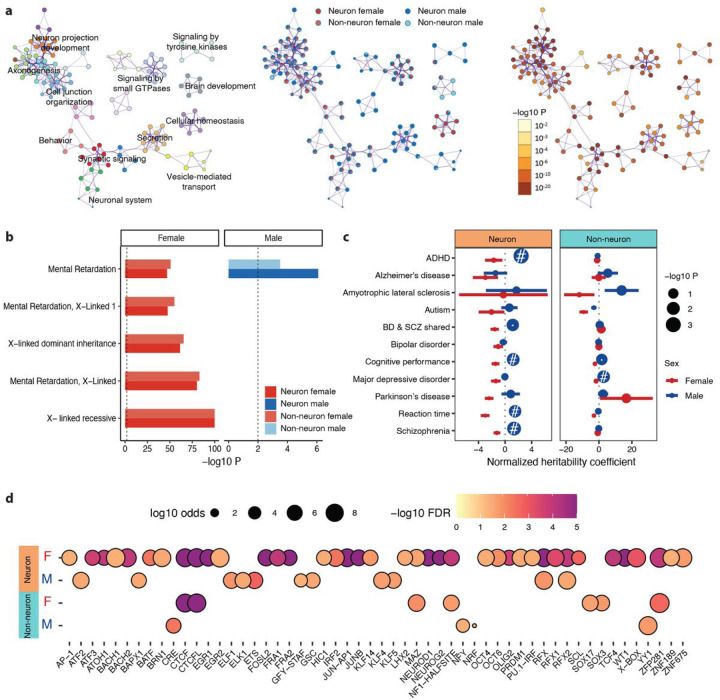
Biological functions and regulatory mechanisms of sex-specific OCRs. **a)** Network plots of functional pathways: (left) colored by cluster ID of enriched terms, with nodes sharing the same cluster ID that are typically close to each other; (middle) represented as pie charts, where pies are colored by gene lists of sex-specific OCRs; (right) colored by p-value, with terms containing more genes generally indicating a more significant p-value. **b)** Bar plot illustrating the significance of top 5 enriched disease gene sets from DisGeNET^33^. Vertical dashed line indicates nominal significance (P-value of 0.01). **c)** Enrichment of trait-associated genetic variants in neuronal and non-neuronal sex-specific OCRs. Horizontal bars indicate standard error of normalized coefficient from LD score regression. Node size is proportional to −log_10_(P-value). # indicates enrichments that are significant at FDR (BH-corrected P-value) < 0.05. **d)** Transcription factor motif enrichment of sex-specific OCRs. Color intensity is proportional to −log_10_ (P-value). Node size is proportional to −log_10_(odds ratio).

To gain insights into the biological pathways regulated by genes associated with sex-specific OCRs, we performed gene set enrichment analysis (GSEA) in each cell type. We identified enrichment in a wide range of biological pathways, including those involved in response to hormones, reproductive processes, metabolism, immune responses, histone modification, and others (Table S4). Specifically, we discovered that genes associated with sex-specific OCRs were involved in synaptic signaling, neuron projection development, and regulation of axonogenesis, which might contribute to the functional sex differences in brain activity (Fig. 2a). Although significantly associated, sex determination, dosage compensation, and regulation of dosage compensation by inactivation of the X chromosome (Table S4) were not ranked among the most enriched pathways. However, we identified X-linked disease terms that were top ranked and significantly associated with female-specific OCRs (Fig. 2b, Table S4). We also identified heparan sulfate (Hs) glycosaminoglycan (GAG) degradation and metabolism in female-specific OCRs, a previously reported sex difference in the hippocampus known to affect neuronal plasticity and brain development^28^. A set of pathways involved in histone modifications, regulated by female-specific OCRs, has been reported to result in sex-specific gene expression (Table S4). Trimethylation of Histone H3 at lysine-36 (H3K36me3), driven by female-specific OCRs, is also observed to be less abundant on the male X chromosome^29^ and is known to induce gene silencing and XCI^30^. Taken together, these results suggest that sex-specific chromatin accessibility plays a critical role in a range of biological pathways, including some that have not previously been linked to sex.

To further explore the importance of sex-specific regulatory mechanisms and their role in disease, we quantified the overlap of sex-specific OCRs with disease risk variants using the LD-score partitioned heritability (LDsc) approach. We note that LDsc does not support analysis on sex chromosomes; therefore, we conducted partitioned heritability analysis using only non-sex chromosomal OCRs. Enrichment of SCZ risk variants was only observed in male-specific neuronal OCRs (Fig. 2c, Table S5). Furthermore, we observed that ADHD, cognitive performance, and reaction time variants were significantly colocalized with male-specific OCRs in neurons. This finding highlights the critical role of sex-specific regulatory mechanisms of neurons in psychiatric disorders and brain functions, specifically in males.

Because transcription factors (TFs) contribute to sex regulating network structures in various human tissues^31^, we hypothesized that TFs can affect patterns of differential chromatin accessibility by differential binding of TF motifs at sex-specific OCRs. We employed TF motif enrichment analysis^32^ in sex-specific OCRs to identify the enrichment of known motifs and to discover novel motifs (Methods). We identified a total of 54 TF motifs enriched for male- and female-specific OCRs (Fig. 2d, Table S6), including 39 TFs enriched for female-specific OCRs and 11 TFs enriched for male-specific OCRs in neurons, as well as 7 TFs enriched for female-specific OCRs and 4 TFs enriched for male-specific OCRs in non-neurons. Of these, 2 neuronal TFs (RFX and RFX2) were shared between males and females, while 5 female-specific TFs (CTCF, CTCFL, MAZ, NF1, and ZFP281) were shared between neurons and non-neurons. We also identified 4 TFs, ELF1, ELK1, KLF5, and YY1, which have been reported to exhibit consistent male-specific enrichment in human tissues^6^. Among these, ELF1, KLF5, and YY1 were enriched in the brain. The impact of sex on the majority of the remaining TFs has not been fully characterized. Overall, these results suggest that a wide range of TFs, some evidently unrelated to sex hormones, play crucial roles in driving sex-specific regulatory programs in the human brain.

### Sex-specific enhancer-promoter regulatory landscape

**Figure 3:**
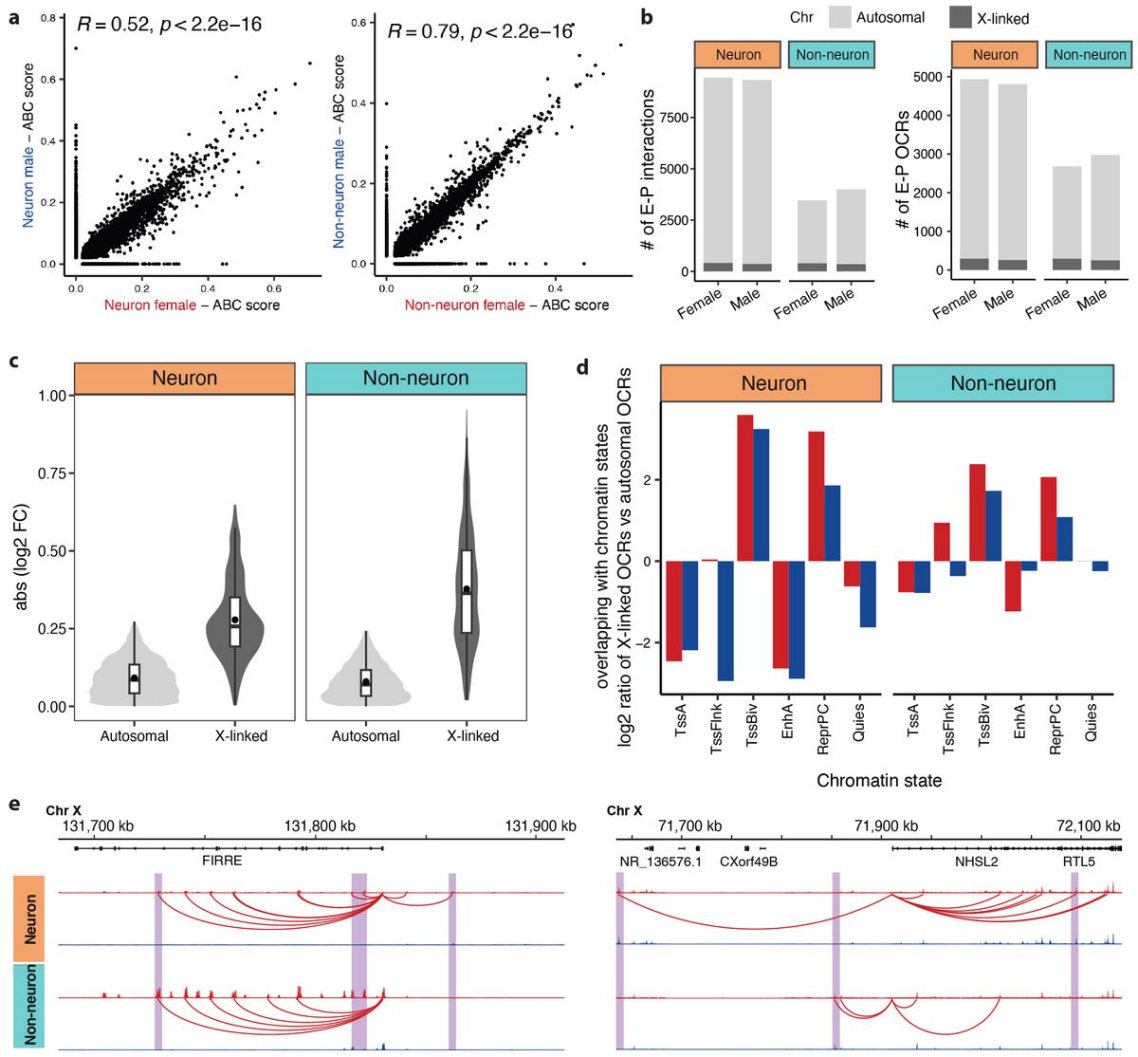
Distal regulatory sex-specific OCRs linked to genes and promoters using the ABC model. **a)** Correlation between ABC scores of E-P_ABC_ interactions from females versus males in neurons (left panel) and non-neurons (right panel). Only interactions with ABC scores exceeding 0.02 in at least one of the compared data sets were used. **b)** Bar plots of the number of sex-specific E-P_ABC_ links and sex-specific OCR_abc_. **c)** Violin plots illustrating the absolute values of log_2_ fold change (FC) distribution of autosomal and X-linked OCR_ABC_ in neurons (left panel) and non-neurons (right panel). Within each violin plot, the box plot provides a summary of the data distribution, with the central black dot indicating the mean, the central horizontal line indicating the median, and the edges of the box indicating the 25th and 75th percentiles. **d)** Log_2_ ratio of relative frequency of overlap with chromatin states for X-linked E-P_ABC_ pairs versus autosomal E-P_ABC_ pairs. EnhA: active enhancers, TssA: active promoters, TssBiv: bivalent promoters, TssFlnk: promoter flanking region, ReprPC: polycomb repression region, and Quies: quiescent region. **e)** Examples of E-P_ABC_ regulatory landscape for sex-specific OCRs regulating XCI genes. The purple shades highlight regions that exhibit cell-type-specific patterns. Female in red; male in blue.

We next sought to determine the concordance between sex-specific transcriptional activity and genome-wide chromatin accessibility. First, to reconstruct functional enhancer-promoter (E-P) pairs responsible for the regulation of sex-specific genes, we applied the activity-by-contact (ABC)^34^ model, combining our open chromatin data, histone acetylation data^35^ and 3D interaction frequency maps^36^. Using separate models for each sex and cell type, we identified 42,119 – 42,229 E-P links (E-P_ABC_) in neurons and 37,772 – 38,261 E-P_ABC_ in non-neurons (Table S7). On average, 64% of E-P_ABC_ were shared between sex in neurons, with a moderate correlation of ABC scores (ρ = 0.52; Fig. 3a left panel), whereas 82% of E-P_ABC_ were shared in non-neurons, with a high correlation score (ρ = 0.79; Fig. 3a right panel). Additionally, when examining these links on autosomal chromosomes versus the X chromosome, we found a significant drop in the correlation between males and females for X-linked genes (Extended Data Fig. 1). This suggests that X-linked gene activity is controlled by sex-specific enhancer activity, aligning with our initial expectations.

Although a majority of enhancers were predicted to interact with a single gene, 38% were linked to two or more genes (Extended Data Fig. 2a). We found that over 70% of the regulated genes were linked to multiple OCRs (OCR_ABC_) (Extended Data Fig. 2b). Of these, only 22% were linked to the nearest gene (Extended Data Fig. 2c). This finding is consistent with our previous analyses^36–38^, demonstrating the limitations of proximity-based annotation of regulatory elements. Still, the frequency of E-P_ABC_ links sharply decreased with distance to the transcription start site (TSS), with 75% of interactions identified within 100 kb, upstream and downstream, of the TSS (Extended Data Fig. 2d).

To evaluate the sex specificity of the observed E-P_ABC_ interactions, we compared sex-specific, but not shared, E-P_ABC_ pairs identified in neurons (9,447 female and 9,332 male pairs) and non-neurons (3,459 female and 3,998 male pairs). On average, 96% of OCR_ABC_ were autosomal and 4% were X-linked in neurons; 90% of OCR_ABC_ were autosomal and 10% were X-linked in non-neurons (Fig. 3b, Table S7). Comparative analysis of sex-specific changes between autosomal and X-linked OCR_ABC_ revealed substantially higher effect sizes for X-linked OCR_ABC_ in both neurons and non-neurons, respectively (Fig. 3c), further confirming that sex-specific effects are predominantly exhibited on the X chromosome on the enhancer level. When compared to adult human brain chromatin states^35^, X-linked OCR_ABC_ regions showed a relative enrichment in polycomb (H3K27me3) repressed chromatin states as opposed to autosomal OCR_ABC_ regions (Fig. 3d, Extended Data Fig. 2e). This observation suggests that X-linked OCR_ABC_ play a role in sex-specific silencing of gene expression.

Moreover, our analysis revealed sex-specific distal regulatory landscapes of XCI in the human brain. We identified 313 E-P_ABC_ regulating 126 genes known to escape XCI (Table S7). For instance, *FIRRE is* an X-linked lncRNA that escapes XCI and is involved in chromosome topological organization^13,27,39^. *FIRRE* harbors a series of putative intronic enhancers demonstrating female-specific chromatin accessibility through differentially regulated E-P_ABC_ (Fig. 3e. Intriguingly, our findings indicate that two enhancers within intron 2 of *FIRRE* are active in male-specific non-neuronal cells, whereas six enhancers spanning introns 2 to 12 are active only in female-specific cells. Notably, there was no observed activity of *FIRRE* enhancers in male-specific neurons. *NHSL2*, a reported XCI gene^6^, is another illustrative example showing that cell-type specific enhancers can regulate sex-specific activity via XCI (Fig. 3e). Taken together, our results indicate that *FIRRE* plays a role in maintaining enrichment of a repressive histone mark (H3K27me3) to mediate gene silencing on the X chromosome. Differential accessibility of putative *FIRRE*s enhancers between neurons and non-neurons further highlights its contribution to cell-type-specific patterns.

### Sex-specific CRDs and TRDs associated with schizophrenia

**Figure 4:**
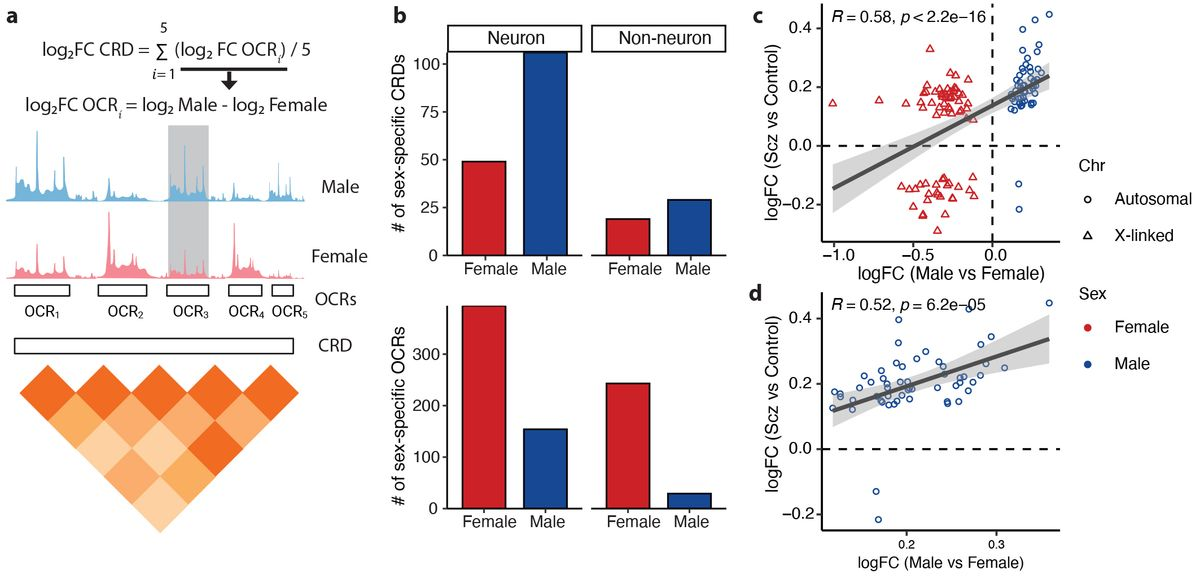
Sex-specific cis-regulatory domains. **a)** Schematic of a CRD obtained from the pairwise correlation of five OCRs. The log_2_FC CRD is summarized as the average of log_2_FC (males versus females) of five OCRs. **b)** Bar plots of the number of sex-specific CRDs (upper panel) and the number of sex-specific OCRs within those CRDs (lower panel). **c)** Correlation of log_2_FC between sex-specific OCRs and SCZ OCRs located within sex-specific CRDs. **d)** Correlation of log_2_FC between male-specific OCRs and SCZ OCRs located within sex-specific CRDs.

*Cis*-regulatory domains (CRDs) are physically interacting regulatory elements that contain multiple locally correlated OCRs and play a crucial role in gene regulation contributing to SCZ risk. Using an in-house analytical framework (Fig. 4a)^15,40^, we showed that 37% of neuronal OCRs were assembled into 6,706 CRDs and 33% of non-neuronal OCRs were assembled into 4,612 CRDs (Extended Data Fig. 3a). Next, we identified 155 neuronal and 48 non-neuronal CRDs that exhibit significant differential accessibility in their mean between males and females (sex-specific CRDs) (Fig. 4b, Table S8). We observed sex-specific OCRs are more likely to be found inside, rather than outside, sex-specific CRDs in both neurons (odds ratio (OR) = 4.39, p-value <2.2e-16) and non-neurons (OR = 7.63, p-value < 2.2e-16) (Extended Data Fig. 3a). We previously identified CRDs that exhibit significantly differential accessibility between individuals with SCZ and controls (SCZ CRDs)^15^. Because OCRs located within neuronal CRDs exhibit significant heritability for SCZ (Extended Data Fig. 3b), we compared the relationship among sex-specific OCRs, SCZ OCRs, sex-specific CRDs and SCZ CRDs in neurons. We found a significant correlation between sex-specific OCRs and SCZ OCRs in neurons, evident in a genome-wide analysis (ρ = 0.59, p-value < 2.2e-16, Extended Data Fig. 4a) and also when focusing on a subset of OCRs inside sex-specific CRDs (ρ = 0.58, p-value < 2.2e-16, Fig. 4c, Extended Data Fig. 4b). The chromatin accessibility of OCRs from male-specific CRDs showed a significant correlation with a SCZ case/control comparison (ρ = 0.52, p-value = 6.2e-5, Fig. 4d, Extended Data Fig. 3c). Conversely, a markedly lower correlation of OCRs from female-specific CRDs (ρ = 0.18, p-value = 0.13) may indicate a different or less direct involvement of these regions with SCZ (Extended Data Fig. 4c, Extended Data Fig. 3c).

**Figure 5:**
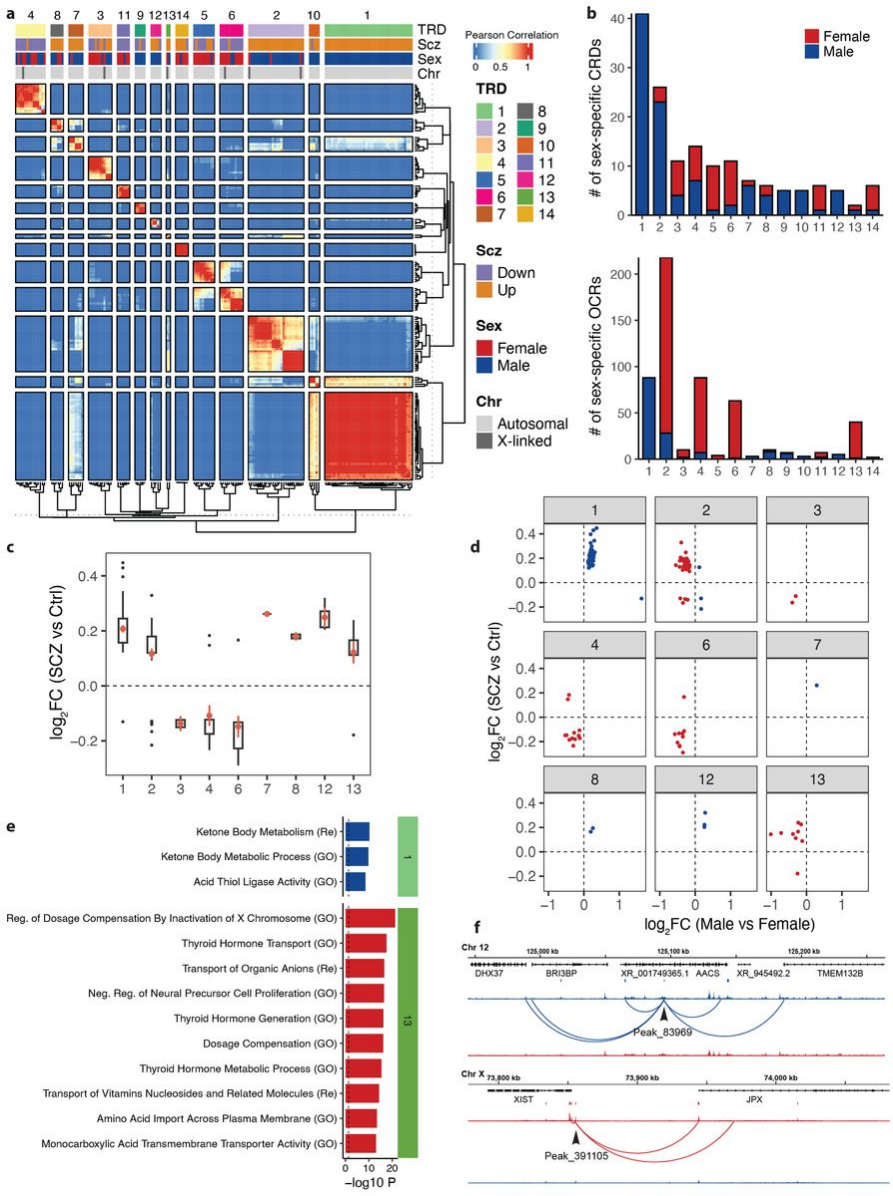
Sex-specific trans-regulatory domains. **a)** Heatmap depicting hierarchical clustering of trans interactions of 155 CRDs that exhibit differential accessibility between males and females in neurons. The layers above the heatmap show: TRD, clustering results in 14 TRDs using gamma statistics; SCZ, upregulation and downregulation of SCZ neuronal CRDs; Sex, female-specific (red) and male-specific (blue) neuronal CRDs; Chr, autosomal (light gray) and X-linked (dark gray) neuronal CRDs. **b)** Bar plots of the number of neuronal sex-specific CRDs per TRD (upper panel) and the number of neuronal sex-specific OCRs per TRD (lower panel). **c)** Boxplot of log_2_FC (SCZ cases vs controls) of neuronal sex-specific OCRs per TRD (TRD5, 9, 10, and 14 are excluded due to lack of sex-specific OCRs (female- and male-specific) within each TRD). **d)** Dot plots of log_2_FC (males vs females) on the x-axis compared to log_2_FC (SCZ cases vs controls) on the y-axis for neuronal sex-specific OCRs per TRD. Female-specific OCRs are in red and male-specific OCRs are in blue. **e)** Functional pathway enrichment of OCRs from neuronal TRD1 and TRD2 that overlap with SCZ-specific OCRs. All enrichments are significant at BH-corrected P-value of 0.05. GO: Gene Ontology, Re: Reactome. **f)** Examples of gene regulatory landscapes for OCRs that show differential chromatin accessibility based on sex (male/female) and SCZ (case/control) status. The upper example illustrates a male-specific OCR in the TRD1 region, while the lower example features a female-specific OCR from the TRD2 region.

We next examined whether the interactions between sex-specific CRDs could identify trans-regulatory domains (TRDs). Using a correlation matrix of expression of sex-specific CRDs across all neuronal samples, we applied hierarchical clustering and Gamma statistics^41^ to identify a total of 14 neuronal sex-specific TRDs (Fig. 5a). While most TRDs exhibit a mix of female- and male-specific CRDs, there is one particular TRD (TRD1) that stands out with a predominant role in the regulation of male-specific CRDs in neurons. TRD1 also showed the highest proportion of male-specific OCRs compared to other TRDs (Fig. 5b). Conversely, TRD2, TRD4, TRD6, and TRD13 displayed a higher proportion of female-specific OCRs (Fig. 5a and b). For TRD1, TRD2, and TRD13, the majority of sex-specific OCRs showed higher accessibility in SCZ cases compared to controls, whereas for TRD4 and TRD6, the majority of sex-specific OCRs showed lower accessibility (Fig. 5c–d). While we observed a moderate correlation of male-specific-with SCZ-changes (ρ = 0.37; p-value = 0.012) in TRD1, we did not observe a significant correlation of female-specific changes with SCZ-changes in any TRD (Extended Data Fig. 5). Thus, this measurement implies sex-specific chromatin regions are likely to be more accessible in SCZ compared to controls. This suggests a complex relationship between chromatin accessibility and the expression of genes associated with SCZ in females, potentially influenced by other regulatory mechanisms or factors^42–44^.

Functional pathway analysis in male-specific OCRs that are upregulated in SCZ and TRD1 identified biological processes related to ketone body metabolism and acid thiol ligase activity (Fig. 5e). These findings are further supported by a recent study elucidating sex-dependent modulation of metabolic hormone^45^, indicating that SCZ may involve sex-specific epigenetic regulation in males, particularly affecting metabolic and enzymatic pathways. Functional pathway analysis in female-specific OCRs that are upregulated in SCZ and TRD13 identified 36 significant associations with biological processes, including regulation of dosage compensation by inactivation of the X chromosome and negative regulation of neural precursor cell proliferation (Fig. 5e; Table S9). This highlights the complexity of female-specific epigenetic regulation in SCZ, particularly emphasizing the role of XCI in neurons^46^. To evaluate the sex specificity of E-P_ABC_ interactions in TRDs, we identified 34 male-specific OCR_abc_ and 95 female-specific OCR_abc_ associated with dysregulation of SCZ in neurons (Table S8). For instance, a male-specific OCR_abc_ (Peak_83969) in TRD1 can regulate four protein-coding genes via E-P_ABC_ interactions, including *TMEM132B, AACS, BRI3BP*, and *DHX37* (Fig. 5f). Similarly, a female-specific OCR_abc_ (Peak_391105) in TRD13 can regulate two lncRNA genes, *XIST* and *JPX* (Fig. 5f), both of which are known XCI genes^21^, suggesting that sex-specific enhancers can regulate activity through XCI within SCZ associated TRDs. This highlights the potential of TRDs as key to understanding the regulatory mechanisms driving sex differences in SCZ.

## Discussion

We have created the largest resource to date, consisting of 1,393 samples derived from neurons and non-neurons, to examine effects of biological sex on chromatin accessibility in the human brain. We first determined differentially accessible regions between males and females (sex-specific OCRs) in both cell types. We found high concordance with sex-specific OCRs from another epigenome study on postmortem brains, confirming the robustness of our findings. As expected, our analysis found the strongest sex bias from regions on the X-chromosome, while the majority of male-specific OCRs regulated autosomal genes, suggesting the impact of sex on regulatory programs throughout the genome. Male- but not female-specific neuronal OCRs were significantly co-localized with common SCZ risk variants and other brain-related traits. Our analysis was restricted to autosomal OCRs due to the inherent limitations of the LD score model. This restriction potentially narrowed our exploration, particularly since sex chromosomes, as documented in the latest SCZ GWAS^47^, are also known to host SCZ loci.

Sex-specific OCRs are involved in a highly diverse set of biological functions. In addition to expected findings, such as dosage compensation by XCI and response to sex hormones, we also observed enrichment for synaptic organization. This is consistent with the role of synapses observed in sex-specific transcriptome studies of SCZ^9^ and depression^48^, and is indicative of their contribution to sex-specific differences in brain development and function. The enrichment of the epigenetic modification H3K36me3 was driven by female-specific OCRs, which is consistent with an established role for this mark in inducing gene silencing involved in XCI^30^. It is important to note that sex-specific H3K36me3 methylation has previously been reported to balance the transcriptional output of the male X chromosome and the fourth chromosome in *Drosophila*^29^. H3K27me3 has also been reported to result in sex-biased gene expression in mammalian placenta^49^ and liver^50^. Conversely, H3K27me3 did not show enrichment within sex-specific OCRs in this study and further investigation integrating histone marks may provide additional mechanistic insights into sex-biased patterns. The large difference in sex-specific chromatin accessibility and enrichment of sex-specific TF motifs between neurons and non-neurons suggests a high degree of cell-type specificity in sex-biased regulatory mechanisms in the human brain.

To date, studies of sex effects on psychiatric and neurologic diseases have largely been limited to the brain transcriptome^6,9,12,48^. In building on this knowledge, our study maps the sex-specific E-P landscape and connects sex-specific OCRs in distal intergenic areas to their target genes. Interestingly, we show that the repressive histone marker, H3K27me3, dependent on the activity of Polycomb repressive complex^51^, exhibited higher levels of X-linked E-P_ABC_ links compared to autosomal E-P_ABC_ links. Our approach enabled us to uncover distal regulatory E-P connections that play a role in governing XCI. To explore the influence of sex on higher-order chromatin structure, we identified *cis*-regulatory domains (CRDs) and trans-regulatory domains (TRDs). Notably, one male-specific TRD (TRD1) showed a significant correlation to SCZ-related changes at the chromatin accessibility level, a pattern not mirrored in the female-specific TRDs (TRD2, TRD4, TRD6, and TRD13). This indicates complex regulatory programs underlying sex differences in SCZ. Beyond gene expression, factors like sex-biased genetic regulation^6^, protein expression^52^, and polygenic risk^43^ may also contribute to phenotypic sex differences in the context of disease. Mapping sex-specific OCRs to E-P_ABC_ links allowed us to pinpoint sex-specific enhancers in TRDs. Intriguingly, among these, certain enhancers were identified that appear to regulate sex-specific activity through XCI in SCZ.

Cumulatively, this study produced maps of sex-specific alterations in the epigenome of both neurons and non-neurons, focusing on changes in chromatin accessibility as well as other higher-order chromosomal conformations. These maps provide valuable insights into the mechanisms underlying sex-specific aspects of SCZ, particularly in relation to XCI. Finally, we provide this unique resource of human brain sex-specific OCRs, E-P links, CRDs and TRDs, enabling the scientific community to further study sex-differentiated mechanisms underlying brain-related disorders.

## Methods

### Study Population

To systematically examine the sex-specific epigenome, we utilized our recently completed atlas of open chromatin accessibility generated using postmortem tissue collected by the CommonMind Consortium (CMC)^53^, as previously described^15,16^. This cohort of SCZ, BD and unaffected controls was assembled after applying stringent inclusion/exclusion criteria. All subjects had to meet the appropriate diagnostic DSM-IV criteria, as determined after review of medical records, direct clinical assessments, and interviews with family members or care providers. Frozen brain tissue derived from ACC (anterior cingulate cortex/Brodmann area 10) and DLPFC (prefrontal cortex/Brodmann area 9 and 46) were obtained from three separate brain banks, including the Icahn School of Medicine at Mount Sinai (MSSM), University of Pittsburgh (PITT) and NIMH Human Brain Collection Core (HBCC). The complete demographic and clinical information of the present study population is provided at the Synapse platform (syn52264219).

### ATAC-Seq Data Generation and Processing

The assay for transposase-accessible chromatin with sequencing (ATAC-seq) was performed using an established protocol^54^ with minor modifications. Throughout the library generation process, randomization was applied with respect to diagnosis status, brain region, and cell type at multiple steps, including indexing and pooling prior to sequencing. ATAC-seq libraries were sequenced by Hi-Seq 2500 and Novaseq 6000 (Illumina) obtaining 50bp paired-end reads. We implemented our in-house pipeline for processing and quality control of ATAC-seq data as described previously^15,16^. The raw reads were trimmed with Trimmomatic^55^ and then mapped to human reference genome GRCh38 with the pseudoautosomal region masked on chromosome Y with the STAR aligner^56^. For each sample, this yielded a BAM file of mapped paired-end reads sorted by genomic coordinates. From these files, reads that mapped to multiple loci, or to the mitochondrial genome, were removed using samtools^57^ and duplicate reads removed with PICARD. Quality control metrics were reported with ataqv^58^, phantomtools^59^ and Picard.

### Differential Chromatin Accessibility

To identify open chromatin regions (OCRs) showing differential accessibility between males and females, we evaluated them statistically. First, we filtered out OCRs that were lowly accessible in most of the samples. Then, we applied an approach based on the Bayesian information criterion to select technical covariates as described in previous publications^15,16^. The final formula for statistical modeling using voom With Dream Weights^17,18^ incorporated both biological covariates, such as sex (i.e., male, female), brain region (i.e., DLPFC, ACC), age at death and diagnosis (i.e., SCZ, BD, and Controls), as well as technical covariates selected by BIC (i.e. fraction of reads in peaks (FRiP) and GC content). The resulting models, based on weighted least-squares linear regression, estimated the effect of the these variables on the chromatin accessibility of each OCR:

- neuronal OCR accessibility ~ sex + diagnosis + brain region + GC_80–100% + FRiP + Fraction_unmapped_reads + GC_20–39/ + AT_dropout + GC_40–59% + Age_of_death + (1 | Individual)
- non-neuronal OCR accessibility ~ sex + diagnosis + brain region + GC_20_39/ + GC_80–100/ + GC_0–19/ + Age_of_death + FRiBP + (11 Individual)

Because we keep multiple samples per specimen, we used the statistical software package dream^17,18^ to properly model correlation structure and thus keep the false discovery rate (FDR) low. Multiple hypothesis testing was adjusted for using an FDR of < 5%.

### Disease Heritability Analysis

To examine the role that identified OCRs may play in various diseases and traits, we tested whether they were enriched in common trait-associated genetic variants from a selection of genome-wide association studies (GWASs)^47,60–69^. For this, linkage disequilibrium (LD) score–partitioned heritability^70^ was used, whereby common genetic variants located in genomic regions of interest are tested to see if they explain more of the heritability than variants not in the regions of interest, while correcting for the number of variants in either category. Additionally, the LD-score approach corrects for potential biases from the general genetic context of the regions of interest, e.g. coding regions and conserved regions. From this regression, a P-value as well as a regression coefficient is outputted. For the AD GWAS, we removed the APOE effect in the model by excluding SNPs around the APOE gene. For MDD and PD GWAS, we used summary statistics generated without 23andMe individuals. The broad MHC-region (hg19:chr6:25–35MB) was excluded due to its extensive and complex LD structure^71^ but, otherwise, default parameters were used.

### Prediction of Enhancer-promoter Interactions

We used the Activity-by-contact model (ABC)^34^ to construct a comprehensive regulatory map of enhancer-promoter (E-P) interactions in neuronal and non-neuronal cell types. This model requires (i) contact frequency between putative enhancers and promoters of regulated genes; and (ii) enhancer activity data. The contact frequency matrices were created from previously generated neuronal and non-neuronal Hi-C datasets composed of eight post-mortem human brains^35^. For enhancer activity data, we used cell type and brain region specific ATAC-seq signal (current study) and H3K27ac ChIP-seq signal^36^. We used the default threshold of ABC score (minimum score of 0.02), the default screening window (5MB around TSS of each gene) and we removed E-P links to ubiquitously expressed genes. Additional information on computational validation of E-P predictions using overlap with chromatin states from the human adult brain can be found in Dong *et. al*^35^.

### *Cis* regulatory landscape analysis

Cell-type specific covariates corrected expression matrices from four datasets (i.e. two cell types and brain regions) were utilized for CRD analysis. The CRD calling pipeline followed the methodology described in Girdhar *et. al*^72^. In total, 6,706 neuronal CRDs and 4,612 non-neuronal CRDs were obtained. We employed a two-stage testing approach using the stageR package^73^ to identify significant CRDs. The process involved two distinct stages: the screening stage (stage 1) and the confirmation stage (stage 2). A CRD was considered sex-specific if it obtained a p-value < 0.05 in stage 2, indicating significant differences in OCR accessibility between males and females. In order to summarize the directionality (or sex specificity) of CRDs, we calculated the mean log_2_FC (males vs females) of OCRs within each CRD. An upregulated (male-specific) CRD had a mean log2FC (males vs females) > 0, while a downregulated (female-specific) CRD had a mean log2FC (males vs females) < 0.

### Clustering of sex-specific CRDs into TRDs

We evaluated whether the interaction between sex-specific CRDs across samples is stratified into domains called trans-regulatory domains (TRDs) that can inform us about specific underlying molecular mechanisms. We limited the identification of TRDs to sex-specific neuronal CRDs because few sex-specific CRDs were observed in non-neurons. The optimal number of clusters was found by evaluating Baker-Hubert GAMMA index^41^ while varying the cluster size (from k = 2:20). GAMMA index is a measure of compactness (how similar are the objects within the same cluster), separation (how distinct are objects from different clusters), and robustness (how reproducible are the clusters in other datasets). Using the GAMMA index, we found k = 14 optimal clusters for neuronal CRDs. Also, we curated two lists, one with all OCRs and the second with only sex-specific OCRs, within each TRD for all downstream analysis. We measured the Spearman correlation coefficient with SCZ-associated changes detected at FDR < 0.05 in Girdhar et. al^72^.

### Gene Set Enrichment and TF Analyses

To explore the function of a gene set, we collected functional gene set annotations from i) MSigDB 7.0 gene sets^74^ of sizes 10 to 1000 genes, ii) SynGO^75^, and iii) DisGeNET database^33^. Fisher exact tests were used to test the enrichment and significance. Metascape was used to perform clustering analysis of significant functional gene sets^76^. To prioritize TFs that are likely responsible for observed changes in sex-specific OCR sets, we performed enrichment analysis using HOMER software (findMotifsGenome.pl)^32^. Significant associations were determined based on an FDR cutoff of 0.05 in each analysis.

## Data Availability

Raw data (FASTQ files) and processed data (BigWig files, peaks, and raw/normalized count matrices) will be available at the time of publication through NIMH Data Archive (https://nda.nih.gov) under collection 5032 (PsychENCODE).
